# Safety and efficacy of single CHAP Hyaluronan injection versus three injections of linear Hyaluronan in pain relief for knee osteoarthritis: a prospective, 52-week follow-up, randomized, evaluator-blinded study

**DOI:** 10.1186/s12891-021-04467-3

**Published:** 2021-06-23

**Authors:** Teng-Le Huang, Chun-Hao Tsai

**Affiliations:** 1 Joint Replacement Center, Jen-Ai hospital, No.483, Dongrong Rd., Dali Dist, Taichung, Taiwan; 2 Department of Orthopedics, China Medical University Hospital, No.91 Hsueh-Shih Road, Taichung, 404 Taiwan; 3 School of Medicine, China Medical University, No.91 Hsueh-Shih Road, Taichung, 404 Taiwan; 4 Department of Sports Medicine, College of Healthcare, China Medical University, Taichung, Taiwan

**Keywords:** WOMAC, Knee osteoarthritis, Hyaluronic acid, Viscosupplementation

## Abstract

**Background:**

The hyaluronic acid (HA) injections are widely used in knee osteoarthritis (OA) patients. We conducted the study comparing the efficacy and safety of single injection of Crosslinked Hyaluronic Acid Platform Hyaluronan (CHAP-HA) with 3-injection of linear hyaluronan in knee OA patients.

**Methods:**

This was a randomized two-arms, evaluator-blinded, controlled, single-center study. Participants with knee OA received single CHAP-HA or three-injection of linear-HA. The 140 patients aged 35–85 years with radiographically confirmed knee OA were enrolled. At week 4, 12, 26, 39, and 52, visual analog scale (VAS) pain score, Western Ontario and McMaster Universities Osteoarthritis (WOMAC) index, timed up and go (TUG) and subject’s adverse events (AE) of these 2 groups were recorded. Primary outcome of the differences of VAS pain score at week 26 between groups was analyzed with analysis of covariance (ANCOVA). At week 52, those who met the inclusion criteria could receive a CHAP-HA injection and being followed-up for the adverse events for 4 weeks.

**Results:**

The trial was conducted from September 2015 to April 2017. A total 140 subjects were available for analysis (71 in the CHAP-HA group and 69 in the linear-HA group). At 26th week, there were significant more improvements in VAS pain scores in CHAP-HA compared with linear-HA. Both CHAP-HA and linear-HA showed significant improvements in the VAS pain score at week 26 compared with the baseline, and the occurrence of adverse events during the study period showed no between-group difference. In subjects with KL = 2, both groups showed significant improvements in VAS pain scores within 26 weeks. In patients with KL = 3, only CHAP-HA group showed significant improvement in VAS pain from 4 to 39 weeks. No unexpected or severe AEs were reported.

**Conclusions:**

A single injection of CHAP-HA may be safe and more effective for 26 weeks in patients with knee OA by comparing to linear-HA; moreover, the pain relief effect of CHAP-HA may remain until 52 weeks. For patients with more severe OA, CHAP-HA was demonstrated to be more preferable to relieve OA pain. Furthermore, repeat treatment of CHAP-HA or using CHAP-HA after a three-injection HA was proved to be safe.

**Trial registration:**

ClinicalTrials.gov: NCT03643588. Date: August 23, 2018 (retrospectively registered).

**Level of Evidence:** Therapeutic Level I.

## Background

Osteoarthritis (OA) is a structural pathology of the joints. It involves proteolysis starting from the edges of joints and cartilage, resulting in lesions, and a decrease in the level of proteoglycans. This pathological change causes cartilage softening and cartilage inflammation [1, 2]. Proteoglycans were found to polymerize with hyaluronic acid (HA) during normal cartilage metabolism but not in degenerated cartilage [3]. Both in vivo and in vitro studies have shown that exogenous HA can induce the polymerization and synthesis of proteoglycans [4, 5], formation of endogenous HA [4], modulation of the inflammatory response [4, 6], reduction of the activity of inflammatory factors, and removal of reactive oxygen species [7]. Intraarticular HA injections can effectively help relieve pain and prevent the deterioration of OA [8–10]. Thus, HA injection is a major option for the treatment of OA pain.

Currently, intraarticular HA treatments are commercially available in five-, three-, and single-injection products. Multiple injections result in a substantial burden to patients because of the time and expense caused by additional hospital visits and the pain associated with each injection. Single-regimen intraarticular HA injection products have therefore become more popular, and their clinical safety and efficacy have been demonstrated [11]. Single-injection regimens involve a higher concentration of HA or antidegradation ability owing to the crosslinked structure of the HA used. Of the various HA crosslinking techniques, use of the crosslinking agent BDDE is safe with a well-established metabolism [12]. One of the BDDE-crosslinked products available in Taiwan is a patented Crosslinked Hyaluronic Acid Platform HA (CHAP-HA). Owing to its crosslinking process, the degradation resistance of CHAP-HA is increased and the molecular weight is elevated to infinity.

There is no clinical trial has focused on the safety and effectiveness between BDDE-crosslinked single-injection products and three-injection products. The present study compared the efficacy and safety over 52 weeks (12 months) of CHAP-HA (HYAJOINT-Plus; SciVision Biotech Inc., Taiwan) with one of the most used three-injection linear HA products in Taiwan (HYALGAN; Fidia Pharma USA, Italy) in patients with knee OA.

## Methods

### Study design

The trial was conducted from September 2015 to April 2017 at China Medical University Hospital, Taichung, in accordance with the International Council for Harmonisation’s Good Clinical Practice Guidelines and the tenets of the Declaration of Helsinki. The study was approved by the China Medical University & Hospital Research Ethics Committee (Approval number: CMUH104-REC2–039) and registered with ClinicalTrials.gov (Identifier: NCT03643588).

This study was a single-center, randomized, two-arm, evaluator-blinded clinical trial. In all, 140 patients with radiographically confirmed knee OA were enrolled in 2015 and 2016. The recruited patients provided written informed consent prior to enrollment. The inclusion criteria were as follows: aged 35–85 years, knee OA pain for 6 months under conventional nonpharmacologic therapy or analgesics, average knee visual analog scale (VAS) pain score ≥ 30 mm on a 100-mm scale, Kellgren–Lawrence (KL) grade 2 or 3 knee OA, and contralateral knee OA pain VAS score < 30 mm. In addition, subjects must be able to understand the study purpose and follow the requirements during the study. The exclusion criteria were as follows: hip OA; KL grade 4 on target knee; leakage, deformity, infection, inflammation, and other active symptoms in the target knee on the injection day; surgery or intraarticular HA injections in the target knee within the past 6 months; intraarticular steroid injections in the target knee within the past 3 months; and allergy to any HA implant. The subjects were asked not to take any analgesic drugs and non-pharmaceutical therapies for the target knee during the study. After applying the inclusion and exclusion criteria, the patients were randomly divided into the CHAP-HA group (single injection, 60 mg/3 mL crosslinked HA) or the linear-HA group (three injections, 20 mg/2 mL). The randomized numbers were generated via Microsoft Excel 2010, and the allocation was performed by one unblinded investigator. Participants took an opaque envelope within a paper labeled with a randomized number, and then the unblinded investigator administered the treatment product according to the allocated intervention. Because patients were aware of their allocations, they were reminded by the blinded study coordinator prior to every visit that they should not inform the evaluator of their allocations.

All patients were followed up by one blinded evaluator for 52 weeks (with visits at 4, 12, 26, 39, and 52 weeks) posttreatment for the evaluation of the safety and efficacy of the treatment.

After the 52-week visit, patients with knee VAS pain scores of ≥30 mm and KL grade of 2 or 3 could choose to receive a single CHAP-HA injection in the target knee. These patients were followed up for another 4 weeks to assess the safety of the repeat treatment and check for cross-reactions between the three-injection and single-injection HA treatments. At weeks 4, 12, 26, 39, and 52, the Western Ontario and McMaster Universities Osteoarthritis Index (WOMAC), VAS pain score, VAS stiffness score, VAS satisfaction score, timed up and go (TUG) test, and adverse events (AEs) were recorded for both groups. The AEs included all the symptoms reported by the patients or signs noticed by the blinded investigator. If an AE was reported by the same patient more than once, the incidence was still counted as 1 and the longer of the two durations was considered. The primary endpoints were the occurrence of any AEs during the study, as well as the subjective assessment of VAS pain at 26 weeks because both products claim 6-month efficacy. A response to treatment was defined as a ≥ 20-mm decrease in the VAS compared with baseline.

### Statistical analysis

SPSS SamplePower 3.0 software (IBM, Armonk, NY, USA) was used to calculate the sample size for this study. Regarding the study purpose, through independent-samples one-way analysis of covariance (ANCOVA) using baseline data for the outcome variable as the covariate, the required sample size was estimated to be 59 participants per group to detect an effect with the power of 0.8 and alpha of 0.05. Because no preliminary data were available, a medium-level Cohen’s d effect size of 0.09 for the R^2^ for the covariate and a medium-level effect size of 0.25 for analysis of variance (ANOVA) were chosen.

All the data were collated into Microsoft Excel 2010 and analyzed with SPSS 12.0 (IBM). The patients’ baseline characteristics in the two groups were compared using independent-sample *t* tests for continuous data or a chi-square test for categorical data. Intention-to-treat analysis was used for the outcome assessments. The last observation carried forward was used for addressing missing data. The differences between baseline and posttreatment within a group were analyzed using repeated-measures one-way ANOVA. Although some posttest data contradicted the normal distribution assumption of independent samples one-way ANCOVA, based on the central limit theory we could obtain the asymptotically robust results from ANCOVA due to large samples. Therefore, ANCOVA and the Bonferroni post hoc test were used to evaluate intergroup difference in follow-up after controlling the baseline data. Subgroup analysis was conducted to assess intergroup differences in VAS pain, WOMAC pain scores, and WOMAC total scores with KL grade 2 or 3; *P* < 0.05 denoted statistical significance.

## Results

### Demographic data

**Fig. 1:**
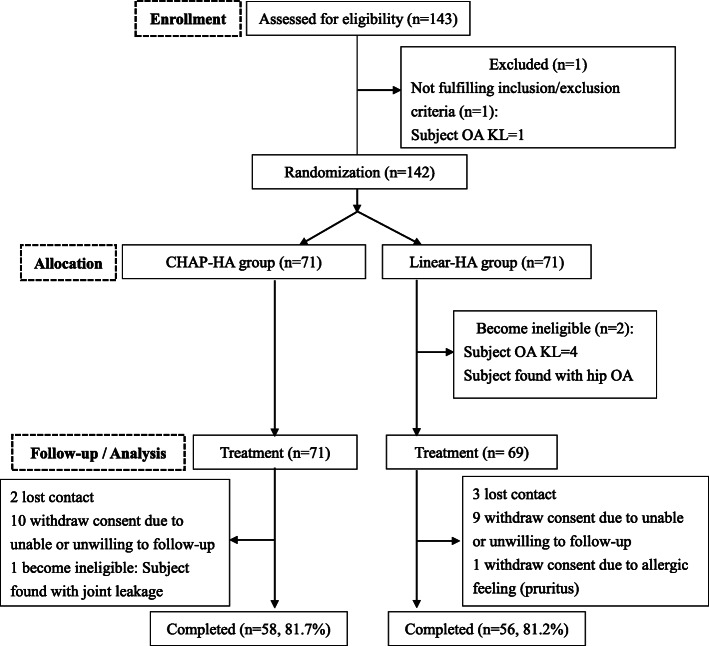
Flow of participants through the trial

A total of 140 patients were enrolled and assigned randomly to one of the two groups (71 in the CHAP-HA group and 69 in the linear-HA group). Total 26 patients (13 patients in each group) dropped out of the study due to lost of their contacts or withdrawal of consent (Fig. 1). Table 1 presents the demographic profile of both groups. No significant differences were observed in patient characteristics between groups. The mean age of the patients was 56.3 years ±11.2 years (range: 36–81 years), with 67.9% women. The sites of injection were evenly divided between the patients. Notably, three-quarters (75.7%) of the patients had OA KL grade 2. Most of the patients had mild- (43.6%) to moderate-level (50.7%) status, and only a few (5.7%) had a heavy work level. The patients’ mean Body Mass Index (BMI) was 25.1 ± 4.0 kg/m^2^. Moreover, 78.9 and 76.8% of patients in the CHAP-HA and linear-HA groups, respectively, received a second injection of CHAP-HA after the 52-week follow-up.

**Table 1:** Demographic data

Variable	Total (*N* = 140)	CHAP-HA (*N* = 71)	Linear-HA (*N* = 69)
Age, years (Mean ± *SD*)	56.3 ± 11.2	56.6 ± 12.6	56.0 ± 9.7
Age group, n (%)			
< 45 years	15 (21.1)	15 (21.1)	12 (17.4)
45–64 years	32 (45.1)	32 (45.1)	41 (59.4)
≥ 65 years	24 (33.8)	24 (33.8)	16 (23.2)
Sex, n (%)			
Male	45 (32.1)	25 (35.2)	20 (29.0)
Female	95 (67.9)	46 (64.8)	50 (71.0)
Site, n (%)			
Left	71 (50.7)	37 (52.1)	34 (49.3)
Right	69 (49.3)	34 (47.9)	35 (50.7)
OA KL grade, n (%)			
2	106 (75.7)	52 (73.2)	54 (78.3)
3	34 (24.3)	19 (26.8)	15 (21.7)
Work status, n (%)			
Mild	61 (43.6)	28 (39.4)	33 (47.8)
Moderate	71 (50.7)	37 (52.1)	34 (49.3)
Heavy	8 (5.7)	6 (8.5)	2 (2.9)
BMI, kg/m^2^	25.1 ± 4.0	25.5 ± 4.2	24.7 ± 3.8
Weight status, n (%)			
Underweight	2 (1.4)	1 (1.4)	1 (1.4)
Normal	59 (42.1)	27 (38.0)	32 (46.4)
Overweight	39 (27.9)	22 (31.0)	17 (24.6)
Obese	40 (28.6)	21 (29.6)	19 (27.5)
Systolic BP (mmHg)	128.5 ± 16.7	129.5 ± 15.9	127.5 ± 17.6
Diastolic BP (mmHg)	74.8 ± 10.6	75.0 ± 9.7	74.5 ± 11.6
Heart rate (beat/min)	74.8 ± 9.1	74.5 ± 9.1	75.2 ± 9.1
Second injection, n (%)			
Non-injection	31 (22.1)	15 (21.1)	16 (23.2)
Left	9 (6.4)	7 (9.9)	2 (2.9)
Right	4 (2.9)	0 (0.0)	4 (5.8)
Both knee	96 (68.6)	49 (69.0)	47 (68.1)

OA KL: Osteoarthritis Kellgren and Lawrence grade

BMI: Body Max Index

BP: Blood pressure

### Efficacy

Table 2 displays the descriptive statistics for the efficacy-related parameters throughout the study period. No significant differences were observed between the baseline values of the dependent variables for the two groups. The VAS pain score was significantly lower in the CHAP-HA group than that in the linear-HA group (21.48 ± 21.51 for CHAP-HA and 37.04 ± 24.51 for linear-HA; *P* < 0.05) with an effect size f of 0.346 at weeks 26; the VAS pain score at weeks 39, and 52 were also significantly lower in the CHAP-HA group than that in the linear-HA group (33.31 ± 20.40 for CHAP-HA and 48.99 ± 21.94 for linear-HA at weeks 39; 43.10 ± 18.45 for CHAP-HA and 51.45 ± 22.33 for linear-HA at weeks 52; *P* < 0.05, *P* = 0.012, respectively) with an effect size f of 0.565, 0.390 after controlling the baseline data as a covariate in ANCOVA. In the CHAP-HA group, the values of VAS pain were significantly lower at all follow-ups compared with baseline (52.90 ± 18.21 at baseline, 25.63 ± 18.44 at weeks 4, 22.42 ± 18.21 at weeks 12, 21.48 ± 21.51 at weeks 26, 33.31 ± 20.40 at weeks 39, and 43.10 ± 18.45 at weeks 52; *P* < 0.05). However, the VAS pain score in the linear-HA group showed a significant reduction compared with baseline was observed only up to 26 weeks (53.19 ± 13.98 at baseline, 29.65 ± 20.56 at weeks 4, 29.39 ± 23.50 at weeks 12, and 37.04 ± 24.51 at weeks 26; *P* < 0.05). VAS stiffness was not significantly different between the groups at any follow-up. Comparing to baseline, both CHAP-HA group and linear-HA group has significantly improved in the VAS stiffness scores until 26 weeks (CHAP-HA: 42.87 ± 22.56 at baseline, 22.61 ± 18.82 at weeks 4, 21.52 ± 20.01 at weeks 12, and 25.52 ± 21.60 at weeks 26; linear-HA: 44.78 ± 20.77 at baseline, 28.58 ± 22.08 at weeks 4, 26.09 ± 19.11 at weeks 12, and 32.97 ± 24.48 at weeks 26; *P* < 0.05). The CHAP-HA group were shown statistical significant improvement in each WOMAC subscore, as well as WOMAC total score compared to the linear-HA group at week 26 and 39 (WOMAC pain subscore: 3.14 ± 3.33 for CHAP-HA and 4.91 ± 2.96 for linear-HA at weeks 26, as well as 4.75 ± 3.47 for CHAP-HA and 6.07 ± 3.16 for linear-HA at weeks 39; WOMAC stiffness subscore: 1.46 ± 1.36 for CHAP-HA and 2.36 ± 1.40 for linear-HA at weeks 26, as well as 1.76 ± 1.53 for CHAP-HA and 2.61 ± 1.58 for linear-HA at weeks 39; WOMAC joint function subscore: 22.37 ± 11.04 for CHAP-HA and 29.99 ± 10.67 for linear-HA at weeks 26, as well as 24.68 ± 11.61 for CHAP-HA and 33.54 ± 11.10 for linear-HA at weeks 39; WOMAC total score: 26.97 ± 15.12 for CHAP-HA and 37.26 ± 14.34 for linear-HA at weeks 26, as well as 31.18 ± 16.03 for CHAP-HA and 42.22 ± 15.05 for linear-HA at weeks 39; *P* < 0.05). In the CHAP-HA group, the values of WOMAC joint function, and WOMAC total scores were significantly lower at all follow-ups compared with baseline (WOMAC joint function: 35.52 ± 11.69 at baseline, 23.27 ± 10.19 at weeks 4, 23.44 ± 10.85 at weeks 12, 22.37 ± 11.04 at weeks 26, 24.68 ± 11.61 at weeks 39, and 30.69 ± 12.62 at weeks 52; WOMAC total scores: 45.80 ± 16.17 at baseline, 28.69 ± 13.94 at weeks 4, 28.35 ± 14.48 at weeks 12, 26.97 ± 15.12 at weeks 26, 31.18 ± 16.03 at weeks 39, and 39.24 ± 17.47 at weeks 52; all *P* < 0.05); while the same results were found in WOMAC pain and WOMAC stiffness except for the values at week 52 (WOMAC pain: 7.34 ± 3.69 at baseline, 3.96 ± 3.31 at weeks 4, 3.49 ± 2.96 at weeks 12, 3.14 ± 3.33 at weeks 26, and 4.75 ± 3.47 at weeks 39; WOMAC stiffness: 2.94 ± 1.67 at baseline, 1.46 ± 1.30 at weeks 4, 1.42 ± 1.23 at weeks 12, 1.46 ± 1.36 at weeks 26, and 1.76 ± 1.53 at weeks 39; *P* < 0.05). In the linear-HA group, a significant reduction in the scores of WOMAC variables was observed until 26 weeks compared with the baseline values (WOMAC pain: 7.17 ± 2.94 at baseline, 3.94 ± 2.49 at weeks 4, 3.43 ± 2.30 at weeks 12, and 4.91 ± 2.96 at weeks 26; WOMAC stiffness: 3.09 ± 1.47 at baseline, 1.88 ± 1.27 at weeks 4, 1.59 ± 1.14 at weeks 12, and 2.36 ± 1.40 at weeks 26; WOMAC joint function: 36.38 ± 10.76 at baseline, 24.83 ± 9.48 at weeks 4, 23.52 ± 8.79 at weeks 12, and 29.99 ± 10.67 at weeks 26; WOMAC total score: 46.64 ± 14.08 at baseline, 30.65 ± 12.39 at weeks 4, 28.55 ± 11.53 at weeks 12, and 37.26 ± 14.34 at weeks 26; *P* < 0.05). A significant lower result was found in the linear-HA group for TUG at weeks 4 when comparing to the CHAP-HA (9.61 ± 1.99 for linear-HA group and 10.95 ± 3.27 for CHAP-HA group; *P* < 0.05). The TUG time showed significant shortened in the CHAP-HA group at week 4, 12 and 26 compared with baseline (11.93 ± 3.65 at baseline, 10.95 ± 3.27 at weeks 4, 10.37 ± 3.21 at weeks 12, and 10.42 ± 3.80 at weeks 26; *P* < 0.05), whereas in the linear-HA groups, participants had significantly better TUG at week 4 and 12 follow-ups compared with baseline (11.05 ± 3.03 at baseline, 9.61 ± 1.99 at weeks 4, and 9.33 ± 2.13 at weeks 12; *P* < 0.05).

**Table 2:** Observational mean and standard deviation of efficacy-related parameters

	CHAP-HA (*N* = 71)			Linear-HA (*N* = 69)			*P*^b^
Variable	Mean	*SD*	*P*^a^	Mean	*SD*	*P*^a^	
VAS pain							
Baseline	52.90	18.21	–	53.19	13.98	–	0.917
4th week	25.63	18.44	< 0.001^‡^	29.65	20.56	< 0.001^‡^	0.216
12th week	22.42	18.21	< 0.001^‡^	29.39	23.50	< 0.001^‡^	0.052
26th week	21.48	21.51	< 0.001^‡^	37.04	24.51	< 0.001^‡^	< 0.001^‡^
39th week	33.31	20.40	< 0.001^‡^	48.99	21.94	1.000	< 0.001^‡^
52th week	43.10	18.45	0.001^‡^	51.45	22.33	1.000	0.012^‡^
VAS stiffness							
Baseline	42.87	22.56	–	44.78	20.77	–	0.603
4th week	22.61	18.82	< 0.001^‡^	28.58	22.08	< 0.001^‡^	0.103
12th week	21.52	20.01	< 0.001^‡^	26.09	19.11	< 0.001^‡^	0.196
26th weeks	25.52	21.60	< 0.001^‡^	32.97	24.48	0.023^‡^	0.069
39th weeks	36.83	20.93	0.555	39.86	22.88	1.000	0.491
52th weeks	41.48	20.88	1.000	44.57	23.21	1.000	0.479
WOMAC pain							
Baseline	7.34	3.69	–	7.17	2.94	–	0.771
4th week	3.96	3.31	< 0.001^‡^	3.94	2.49	< 0.001^‡^	0.952
12th week	3.49	2.96	< 0.001^‡^	3.43	2.30	< 0.001^‡^	0.944
26th week	3.14	3.33	< 0.001^‡^	4.91	2.96	< 0.001^‡^	< 0.001^‡^
39th week	4.75	3.47	< 0.001^‡^	6.07	3.16	0.360	0.014^‡^
52th week	5.96	3.84	0.117	6.84	3.88	1.000	0.126
WOMAC stiffness							
Baseline	2.94	1.67	–	3.09	1.47	–	0.592
4th week	1.46	1.30	< 0.001^‡^	1.88	1.27	< 0.001^‡^	0.061
12th week	1.42	1.23	< 0.001^‡^	1.59	1.14	< 0.001^‡^	0.454
26th week	1.46	1.36	< 0.001^‡^	2.36	1.40	0.020^‡^	< 0.001^‡^
39th week	1.76	1.53	< 0.001^‡^	2.61	1.58	0.544	0.002^‡^
52th week	2.59	1.57	1.000	2.83	1.68	1.000	0.477
WOMAC joint function							
Baseline	35.52	11.69	–	36.38	10.76	–	0.653
4th week	23.27	10.19	< 0.001^‡^	24.83	9.48	< 0.001^‡^	0.415
12th week	23.44	10.85	< 0.001^‡^	23.52	8.79	< 0.001^‡^	0.906
26th week	22.37	11.04	< 0.001^‡^	29.99	10.67	0.003^‡^	< 0.001^‡^
39th week	24.68	11.61	< 0.001^‡^	33.54	11.10	1.000	< 0.001^‡^
52th week	30.69	12.62	0.030^‡^	36.67	12.98	1.000	0.006^‡^
WOMAC total score							
Baseline	45.80	16.17	–	46.64	14.08	–	0.745
4th week	28.69	13.94	< 0.001^‡^	30.65	12.39	< 0.001^‡^	0.414
12th week	28.35	14.48	< 0.001^‡^	28.55	11.53	< 0.001^‡^	0.986
26th week	26.97	15.12	< 0.001^‡^	37.26	14.34	< 0.001^‡^	< 0.001^‡^
39th week	31.18	16.03	< 0.001^‡^	42.22	15.05	0.711	< 0.001^‡^
52th week	39.24	17.47	0.044^‡^	46.33	17.91	1.000	0.016^‡^
TUG (second)							
Baseline	11.93	3.65	–	11.05	3.03	–	0.120
4th week	10.95	3.27	0.018^‡^	9.61	1.99	< 0.001^‡^	0.009^‡^
12th week	10.37	3.21	< 0.001^‡^	9.33	2.13	< 0.001^‡^	0.108
26th week	10.42	3.80	0.006^‡^	10.30	2.35	0.091	0.372
39th week	10.91	3.83	0.159	10.78	2.22	1.000	0.374
52th week	11.59	4.50	1.000	11.35	2.27	1.000	0.657

VAS: Visual Analogue Scale

WOMAC: Western Ontario and McMaster Universities Arthritis Index

TUG: Timed Up and Go Test

a, corrected *P* value between post-injections and baseline;

b, *P* value between groups;

‡, significant, *P* < 0.05

The response rate in the CHAP-HA group was significantly higher than that in the linear-HA group at weeks 26 (74.6% vs. 44.9%, respectively, *P* < 0.001) and 52 (38.0% vs. 18.8%, respectively, *P* = 0.012).

No significant differences were observed in terms of satisfaction between the two groups at weeks 4 and 12 (Table 3), but patients began to significantly favor treatment with CHAP-HA from week 26 until the end of the study (73.4 ± 22.7 for CHAP-HA and 63.5 ± 26.5 for linear-HA at weeks 26; 72.3 ± 22.4 for CHAP-HA and 52.1 ± 23.2 for linear-HA at weeks 39; 61.7 ± 22.0 for CHAP-HA and 37.5 ± 23.1 for linear-HA at weeks 52; *P* < 0.05). The CHAP-HA group reported an overall over 60% satisfaction at week 52. The linear-HA group reported about 60% satisfication at week 26, but then the satisfication gradually decreased at week 39 and 52 (52.1 and 37.5%, respectively).

**Table 3:** Patient satisfaction in time interval

Time	CHAP-HA (*N* = 71)	Linear-HA (*N* = 69)	*P* value
4th week	66.4 ± 22.4	68.4 ± 24.7	0.622
12th week	73.2 ± 23.4	71.1 ± 25.2	0.601
26th week	73.4 ± 22.7	63.5 ± 26.5	< 0.018^‡^
39th week	72.3 ± 22.4	52.1 ± 23.2	< 0.001^‡^
52th week	61.7 ± 22.0	37.5 ± 23.1	< 0.001^‡^

‡ indicates a significant difference between groups (*P* < 0.05)

Table 4 shows the VAS pain of both products for different levels of OA severity. In the CHAP-HA group, the improvements within 26 weeks achieved a significant level in both KL grades 2 and 3 (19.96 ± 20.93 for CHAP-HA and 32.70 ± 23.02 for linear-HA in KL grades 2; 25.63 ± 23.09 for CHAP-HA and 52.67 ± 24.04 for linear-HA in KL grades 3 at weeks 26; *P* < 0.05). However, in the linear-HA group, patients with KL grade 3 did not appear to benefit as much as those with KL 2 grade. Among patients with KL grade 3 OA, those in the CHAP-HA group exhibited significantly higher pain relief than those in the linear-HA group in an earlier treatment time (1–6 months), but among those with KL grade 2 OA, the significant difference was found at a later treatment time (6–12 months; *P* < 0.05). Posterior powers of VAS pain were above 0.8 at week 26 and 39 in KL grade 2 as well as at week 4, 12, and 26 in KL grade 3 similarly. Because of the small sample number of the subjects, there were some data not normal distributed in OA KL = 3. The data of week 4, 26, and 52 for CHAP-HA group were not normal distributed (median: 20.00, interquartile range (IQR): 40.00 at 4-week follow-up; median: 20.00, IQR: 35.00 at 26-week follow-up; median: 45.00, IQR: 45.00 at 52-week follow-up). Aside from CHAP-HA group, the data of linear-HA group in OA KL = 3 were normal distributed at each timepoint. Other than VAS pain, it is also found that in the WOMAC pain subscore in KL grade 2 subgroup analysis, the CHAP-HA group had significant improvement comparing to linear-HA group at week 26 (2.73 ± 3.31 for CHAP-HA, 4.61 ± 2.88 for linear-HA, *P* = 0.002; posterior powers > 0.8). In WOMAC total score, between group comparison had shown significantly reduced in CHAP-HA with KL grade 2 at week 26 as well as 39 (CHAP-HA: 24.33 ± 14.46, linear-HA: 35.30 ± 13.52, *P* < 0.05 at week 26; CHAP-HA: 29.94 ± 15.60, linear-HA: 39.87 ± 14.30, *P* = 0.001 at week 39; posterior powers > 0.8, respectively). Also, in KL grade 3 at week 39, the WOMAC total score for CHAP-HA was 34.58 ± 17.12, lower than 50.67 ± 15.14 of linear-HA group (*P* = 0.002; posterior powers > 0.8).

**Table 4:** The comparison between groups according to Osteoarthritis Kellgren and Lawrence grade

	OA KL = 2							OA KL = 3						
Variables	CHAP-HA (*N* = 52)			Linear-HA (*N* = 54)			*P*^b^	CHAP-HA (*N* = 19)			Linear-HA (*N* = 15)			*P*^b^
	Mean	*SD*	*P*^a^	Mean	*SD*	*P*^a^		Mean	*SD*	*P*^a^	Mean	*SD*	*P*^a^	
VAS pain														
Baseline	51.50	19.30	–	50.19	13.53		0.685	56.74	14.59		64.00	9.86		0.109
4th week	27.02	17.94	< 0.001^‡^	24.65	19.02	< 0.001^‡^	0.579	21.84	19.74	< 0.001^‡^	47.67	15.45	0.031^‡^	0.001^‡^
12th week	21.58	17.12	< 0.001^‡^	23.57	20.53	< 0.001^‡^	0.591	24.74	21.24	< 0.001^‡^	50.33	22.08	0.497	0.007^‡^
26th week	19.96	20.93	< 0.001^‡^	32.70	23.02	< 0.001^‡^	0.003^‡^	25.63	23.09	< 0.001^‡^	52.67	24.04	0.934	0.009^‡^
39th week	30.38	18.88	< 0.001^‡^	46.85	21.40	1.000	< 0.001^‡^	41.32	22.72	0.059	56.67	22.89	1.000	0.241
52th week	41.25	18.36	0.012^‡^	48.89	21.99	1.000	0.034^‡^	48.16	18.20	0.225	60.33	22.24	1.000	0.389

OA KL: Osteoarthritis Kellgren and Lawrence grade

VAS: Visual Analogue Scale

a, corrected *P* value between post-injections and baseline;

b, *P* value between groups;

‡, significant, *P* < 0.05

### Adverse events

Table 5 lists the AEs that occurred at the time of the first injection in each study group. No unexpected or severe AEs were reported. The most frequent AEs recorded in the CHAP-HA group were joint pain (29.6%), joint swelling (19.7%), paresthesia (2.8%), and stiffness (1.4%). The most frequent AEs recorded in the linear-HA group were joint pain (18.8%), pruritus (1.4%), joint swelling (8.7%), and paresthesia (2.9%). No significant differences were noted between the groups in terms of AEs. Table 5 also summarizes the AEs that occurred at the time of the second injection of CHAP-HA. Both CHAP-HA and linear-HA groups reported joint pain (4.2% vs. 4.3%), joint swelling (2.8% vs. 1.4%), and paresthesia (2.8% vs. 4.3%). Most of the AEs were resolved within 1–2 weeks.

**Table 5:** Adverse event at injection

Adverse event	CHAP-HA	Linear-HA	*P* value
First Injection	(*N* = 71)	(*N* = 69)	
Joint swelling	14 (19.7)	6 (8.7)	0.062
0–3 days	3	4	
4–7 days	7	2	
8–14 days	3	0	
> 14 days	1	0	
Joint pain	21 (29.6)	13 (18.8)	0.139
0–3 days	7	4	
4–7 days	6	6	
8–14 days	7	0	
> 14 days	1	3	
Joint disorder	6 (8.5)	1 (1.4)	0.057
0–3 days	3	1	
4–7 days	2	0	
8–14 days	0	0	
> 14 days	1	0	
Pruritus	0 (0.0)	1 (1.4)	0.493
0–3 days	0	1	
Paraesthesia	2 (2.8)	2 (2.9)	0.977
0–3 days	1	1	
8–14 days	1	1	
Pain in limb	1 (1.4)	0 (0.0)	0.322
8–14 days	1	0	
Myasthenia	1 (1.4)	0 (0.0)	0.322
4–7 days	1	0	
Fatigue	2 (2.8)	0 (0.0)	0.160
0–3 days	2	0	
Stiffness	1 (1.4)	0 (0.0)	0.322
0–3 days	1	0	
Sore	1 (1.4)	2 (2.9)	0.543
0–3 days	1	2	
Second Injection	(*N* = 56)	(*N* = 53)	*P* value
Joint swelling	2 (2.8)	1 (1.4)	0.576
0–3 days	1	0	
4–7 days	1	0	
> 14 days	0	1	
Joint pain	3 (4.2)	3 (4.3)	0.971
0–3 days	3	0	
4–7 days	0	3	
Paraesthesia	2 (2.8)	3 (4.3)	0.626
0–3 days	0	1	
4–7 days	2	2	
Joint disorder	1 (1.4)	0 (0.0)	0.322
4–7 days	1	0	

The values are given as the number of patients with the percentage in parentheses

## Discussion

The results of the present study reflect the efficacy of a single-dose HA injection in the treatment of knee OA. The observed reduction in pain was clinically relevant in this study because a reduction in pain intensity ≥30% means clinical difference in knee OA [13, 14]. In addition, the result showed that CHAP-HA was clinically superior to linear-HA based on a medium to large effect size (> 0.25). The accepted threshold for clinically relevant improvement in pain based on VAS score is ≥10–30 mm and is 14 mm on average [15]. In the present study, response to treatment was considered with VAS pain score improvement of ≥20 mm; accordingly, the response was significantly higher at weeks 26 and 52 in the CHAP-HA group than it was in the linear-HA group, especially at week 26. Thus, BDDE-crosslinked HA can achieve clinically significant pain relief for knee OA. The within-group analysis also revealed improvements in VAS pain and WOMAC total scores consistently over 52 weeks in patients who received BDDE-crosslinked HA, indicating that the treatment might be beneficial for longer than the claim (6 months). However, such an indication remains unclear and requires further research.

In this trial, the recorded AEs were mild and self-limiting. Most of the AEs were pain and swelling and were comparable between the two HA injections, indicating that both treatment options are safe. Most treatment-related AEs were reported within 2 days after injection in both groups. However, the incidence was slightly higher in patients treated with BDDE-crosslinked HA. It has been reported that approximately 20% of patients develop arthralgia after intraarticular HA injection [16–18]. One 26-week, multicenter, randomized, double-blinded study demonstrated that the rates of knee joint pain in patients treated with a single injection of NASHA, which is also a BDDE-crosslinked HA, and those treated with saline were 6.4 and 2.9%, respectively [19]. In a 26-week clinical trial, the incidence of AEs was slightly higher in patients receiving single 6-mL intraarticular hylan G-F 20 compared with in those receiving placebo (5.7% vs. 3.1%) [20]. Althought both studies showed no statistically significance, it seems that crosslinked HA might cause a higher incidence of AEs, even though patients could tolerate these discomforts because of their overall effects. Lower incidence of AEs may explain the higher satisfaction with linear HA in the first 4 weeks follow-up, but the satisfaction with BDDE-crosslinked HA increased later in our study. The mechanism has not been clarified but higher occurance of transient local reactions were inferred to result from the injection procedure which might be owing to the higher extrusion pressue associated with the viscous property of crosslinked HA [21–23]. Nevertheless, patients should be warned of these side-effects when choosing these products. In the group receiving the second injection of hylan G-F 20, one patient (1.3%) experienced treatment-related AEs [20]. In the present study, the incidence of AEs with the second injection of CHAP-HA was lower than that with the first injections, implying that patients may have become aware of these AEs and found them more tolerable. Furthermore, the incidence of AEs in patients who received linear HA and BDDE-crosslinked HA were similar to those who received BDDE-crosslinked HA twice, indicating no cross-reactions between the two products.

This study also found that pain relief with BDDE-crosslinked HA was significantly better than that with linear HA from week 26. Several studies have demonstrated the higher the molecular weight of the synovial fluid supplement, the greater their effectiveness, which probably depends on the improved viscoelastic properties and antiinflammation ability [24–26]. However, the present study lacked a placebo control, and thus the efficacy of the crosslinked HA beyond 6 months requires more clinical evidence.

The study also found that patients with higher KL grade seemed to obtain an earlier pain relief benefit with the BDDE-crosslinked HA treatment, whereas those with lower KL grade obtained the benefits relatively later. This might be correlated with a higher incidence of local discomforts, particularly in patients with lower KL grade, because of the higher viscosity or lower fluidity of the product. Thus, patients with better OA conditions may not obtain considerable benefits from BDDE-crosslinked HA in the beginning. As the product degrades, HA may modulate biological factors to achieve its pain reduction effects. The pain reduction effect of HA is likely mediated through interaction with cellular receptors, such as opioid receptors, nociceptors, and CD44 [5, 27–29], and the initiation of further signaling pathways of PGE_2_ downregulation and COX-2 generation [27, 29]. Although those studies have focused on linear products, crosslinked products degrade into a linear form over time, and owing to the longer degradation time, these pain-relieving effects may last longer. On the other hand, patients with higher KL grade had better and earlier pain relief likely due to the very low amount of viscofluid in the joints, thereby allowing the highly viscous BDDE-crosslinked HA to achieve its intended treatment from the beginning.

The present study had several limitations. First, we did not include a placebo treatment (i.e., saline injection), and thus we could not clinically confirm the > 6-month effectiveness of BDDE-crosslinked HA. Second, we did not recruit patients with KL grade 4 OA. Third, because of the comparison between single and three injections, patients could not be blinded, which may have led to subjective outcome bias. Finally, We performed various analyses on many parameters, but we focused the results on primary endpoint VAS. However, the problem of increased type I error due to multiple testings for the differences in other secondary parameters should be cautioned.

## Conclusions

In conclusion, our results demonstrated clinical improvement in both pain and function with BDDE-crosslinked HA lasting for 52 weeks, and the improvement was preferable compared with linear HA from 6 to 12 months. In the future, we hope to recruit patients with KL grade 4 OA to investigate the effects of intraarticular injection of crosslinked HA in that group and to more accurately identify patients who can benefit from this treatment.

## Data Availability

The datasets used and/or analysed during the current study are available from the corresponding author on reasonable request.

## Abbreviations

HA: Hyaluronic acid
CHAP: Crosslinked Hyaluronic Acid Platform
OA: Osteoarthritis
VAS: visual analog scale
KL: Kellgren–Lawrence
WOMAC: Western Ontario and McMaster Universities Osteoarthritis Index
TUG: Timed up and go test
AE: Adverse event
ANCOVA: One-way analysis of covariance
IQR: Interquartile range

## Declarations

The study adheres to CONSORT guidelines.
